# Temporal Trends and Lesion Sets for Persistent Atrial Fibrillation Ablation: A Meta-Analysis With Trial Sequential Analysis and Meta-Regression

**DOI:** 10.1161/CIRCEP.123.011861

**Published:** 2023-08-17

**Authors:** Arunashis Sau, Sharan Kapadia, Sayed Al-Aidarous, James Howard, Afzal Sohaib, Markus B. Sikkel, Ahran Arnold, Jonathan W. Waks, Daniel B. Kramer, Nicholas S. Peters, Fu Siong Ng

**Affiliations:** National Heart and Lung Institute, Imperial College London, United Kingdom (A. Sau, S.K., J.H., M.B.S., A.A., D.B.K., N.S.P., F.S.N.).; Department of Cardiology, Imperial College Healthcare NHS Trust, London, United Kingdom (A. Sau, J.H., A.A., N.S.P., F.S.N.).; UCL Institute of Cardiovascular Science, University College London, United Kingdom (S.A.-A.).; The Barts Heart Centre, St Bartholomew’s Hospital, Barts Health NHS Trust, London, United Kingdom (A. Sohaib).; Royal Jubilee Hospital, Victoria, Canada (M.B.S.).; Harvard-Thorndike Electrophysiology Institute, Beth Israel Deaconess Medical Center, Harvard Medical School, Boston, MA (J.W.W.).; Richard A. and Susan F. Smith Center for Outcomes Research in Cardiology, Beth Israel Deaconess Medical Center, Harvard Medical School, Boston, MA (D.B.K.).

**Keywords:** atrial fibrillation, meta-analysis, randomized controlled trials

## Abstract

**BACKGROUND:**

Ablation for persistent atrial fibrillation (PsAF) has been performed for over 20 years, although success rates have remained modest. Several adjunctive lesion sets have been studied but none have become standard of practice. We sought to describe how the efficacy of ablation for PsAF has evolved in this time period with a focus on the effect of adjunctive ablation strategies.

**METHODS:**

Databases were searched for prospective studies of PsAF ablation. We performed meta-regression and trial sequential analysis.

**RESULTS:**

A total of 99 studies (15 424 patients) were included. Ablation for PsAF achieved the primary outcome (freedom of atrial fibrillation/atrial tachycardia rate at 12 months follow-up) in 48.2% (5% CI, 44.0–52.3). Meta-regression showed freedom from atrial arrhythmia at 12 months has improved over time, while procedure time and fluoroscopy time have significantly reduced. Through the use of cumulative meta-analyses and trial sequential analysis, we show that some ablation strategies may initially seem promising, but after several randomized controlled trials may be found to be ineffective. Trial sequential analysis showed that complex fractionated atrial electrogram ablation is ineffective and further study of this treatment would be futile, while posterior wall isolation currently does not have sufficient evidence for routine use in PsAF ablation.

**CONCLUSIONS:**

Overall success rates from PsAF ablation and procedure/fluoroscopy times have improved over time. However, no adjunctive lesion set, in addition to pulmonary vein isolation, has been conclusively demonstrated to be beneficial. Through the use of trial sequential analysis, we highlight the importance of adequately powered randomized controlled trials, to avoid reaching premature conclusions, before widespread adoption of novel therapies.

**WHAT IS KNOWN?**

- Although ablation for persistent atrial fibrillation has been performed for over 20 years, success rates have remained modest.
- Several adjunctive lesion sets, on top of pulmonary vein isolation, have been studied but none have become standard of practice.

**WHAT THE STUDY ADDS**

- We found that overall success rates from persistent atrial fibrillation ablation and procedure/fluoroscopy times have improved over time.
- However, no adjunctive lesion set, in addition to pulmonary vein isolation, has been conclusively demonstrated to be beneficial.
- Through the use of trial sequential analysis, we were able to highlight the importance of adequately powered randomized controlled trials, to avoid reaching premature conclusions.

Pulmonary vein isolation (PVI) was first described as a treatment for atrial fibrillation (AF) ≈2 decades ago,^1^ following the identification of pulmonary vein triggers for AF by Haissaguerre and colleagues^2^ in 1998. PVI has since become the mainstay in the treatment of paroxysmal AF,^3^ and could be considered as the first-line therapy in some patients.^4,5^ In the last 20 years, there has been significant evolution in the technology for PVI leading to greater safety and effectiveness. This includes improvements to radiofrequency point-by-point ablation in the form of irrigated catheters, contact force and three-dimensional (3D) electroanatomical mapping, and the invention of single shot techniques such as the cryoballoon.^6^ Despite these advances, ablation for persistent AF (PsAF) has limited efficacy compared with paroxysmal AF, with single procedure success rates in the region of 43%.^7^

In an attempt to improve these success rates, many adjunctive ablation strategies have been studied, including complex fractionated atrial electrogram (CFAE) ablation, linear ablation, posterior wall isolation (PWI) isolation, and driver ablation,^8^ although none have become universally accepted, and each introduces potential risks. With each of the adjunctive ablation strategies, there was initial early promise with high success rates from the initial single-center studies, leading to high uptake.^9^ However, the initial reports of high efficacy for adjunctive ablation strategies were not confirmed at subsequent randomized controlled trials (RCTs),^10^ and the enthusiasm for adjunctive ablation has since waned.^9^

Conducting adequately powered RCTs of adjunctive ablation strategies is challenging, and the interpretation of data on adjunctive ablation has often relied on meta-analyses of multiple RCTs, which is generally considered the top of the hierarchy of evidence.^11^ However, meta-analysis has its own limitations, in particular, a risk of type I error. It has been shown that 7% of Cochrane reviews have made false positive conclusions. Importantly, it would have been possible to avoid 93% of these false positives through the use of trial sequential analysis (TSA).^12^ TSA handles meta-analysis of several RCTs in a manner similar to interim analysis of an RCT. If the required information size has not been reached, the uncertainty surrounding the estimate of the intervention effect will increase, thereby reducing the likelihood of a type I error.^13^

In this study, we sought to describe how the efficacy of ablation for PsAF has evolved in the last 2 decades, with a focus on the effect of adjunctive ablation strategies. In particular, we aimed to evaluate the past and current landscape and assess the need for future studies of each additional ablation strategy. We performed a systematic review with meta-regression and cumulative meta-analysis to address these aims. Additionally, we used TSA to investigate how premature conclusions can be avoided when evaluating the efficacy of novel ablation strategies.

## METHODS

The data that support the findings of this study are available from the corresponding author, F.S.N., on reasonable request.

This study uses only summary data from previously published studies, ethical approval was therefore not required.

### Search Strategy

The MEDLINE and Cochrane Central Register of Controlled Trials were searched for studies of PsAF ablation until June 16, 2022. To include more recent studies after the original search, an expanded search was performed until April 21, 2023. The search string included (persistent or nonparoxysmal or non paroxysmal) AND atrial fibrillation AND ablation AND the Cochrane high sensitive search strategy for MEDLINE.^14^ Abstracts and relevant full texts were screened by the reviewers (A.S., S.K., and S.A.); any disputes were resolved by consensus following discussion with another author (M.S. or F.S.N.). The review protocol of this study was published in the PROSPERO (International Prospective Register of Systematic Reviews; CRD42022341807) database.

### Inclusion and Exclusion Criteria

Study inclusion criteria were similar to our previous study^8^: (1) randomized and prospective nonrandomized trials published in English; (2) patient population with PsAF or long-standing persistent atrial fibrillation; (3) at least 1 intervention arm including some form of endocardial left atrial ablation; (4) minimum follow-up period of 3 months; and (5) outcome measure of freedom from AF or freedom from AF/atrial arrhythmia after a single procedure. Studies of patient cohorts or subsets not representative of the general population with AF (eg, only patients with heart failure, diabetes, or obesity) were excluded.

For this analysis, given the pivotal role of PVI in PsAF ablation, study arms without PVI were excluded. Outcomes at the 12-month time point were reported in the majority of studies and was felt to be clinically relevant as most recurrences would be expected to occur by this point; therefore, analyses were performed on this group of studies.

### Data Extraction

Three authors (A.S., S.K., and S.A.) extracted the data from the included studies. Where possible, continuous variables were extracted as mean±SD, and categorical variables were taken as percentages.

### Outcomes

The primary outcome was percentage of patients free from AF/atrial tachycardia (AT) after a single procedure at 12 months. Studies with follow-up <12 months were used only for procedure and fluoroscopy times and complications data. Data were taken primarily from study text or tables. Patient-level data were not available publicly and not practical to obtain from the large number of studies included. Where a 12-month outcome was not reported in the text or tables, this data was extracted from the KM curve. Studies that did not report an end point of freedom from AF/AT at 12 months were not included in this analysis. Major complications were defined as transient ischemic attack, stroke, tamponade, atrioesophageal fistula, and death.

### Analysis

Percentage freedom from recurrence of AF/AT was used as the dependent variable, and a meta-regression was performed using the restricted maximum likelihood estimator, with study-level heterogeneity factored using a random-effects model. The statistical programming environment R with the metafor package was used for all statistical analysis.^15^ The PRISMA 2020 guideline was used to report results.^16^

A cumulative meta-analysis was performed, where studies were added one-by-one in order of publication year and month, and a meta-analysis was performed after the addition of each study.^17^ Random-effects meta-analysis was performed using the restricted maximum likelihood estimator. The I2 statistic was used to assess heterogeneity.

TSA can be used to better control meta-analyses for type 1 and type 2 errors.^13^ TSA was performed using TSA program version 0.9.5.10 beta.^18^ We pragmatically anticipated an intervention effect of 20% and performed additional sensitivity analyses at 15%, which is consistent with the anticipated relative risk reduction used to power CAPLA.^19^ It is important to note the results of TSA are sensitive to the choice of intervention effect. We used a random-effects model with CIs of 95%, an information axis with sample size, type 1 error of 2-sided boundary type of 5%, and power of 80%.

Publication bias was assessed through visual inspection of a funnel plot. Risk of bias for the RCTs included in the meta-analyses was assessed with the Cochrane risk of bias tool.^20^

## RESULTS

Searches yielded a total of 2186 studies. A total of 99 studies (184 study arms) were included with a total of 15 424 patients. A study screening flowchart is shown in Figure S1. Average age was 61.0±3.8 years and 74.0±11.9% were male. A total of 66 studies were RCTs, and the remainder (33) were prospective observational studies. The first included study was published in 2006, and the most recent in 2023. The funnel plot is shown in Figure S2, and there was no visual evidence of publication bias.

We have previously described meta-regression interpretation.^8^ Briefly, meta-regression aims to explain the outcome by a variety of variables. The effect of each variable is separated from the others in the model.

### Overall Outcomes Following Ablation for PsAF

Including all study arms, ablation for PsAF achieved the primary outcome (freedom of AF/AT rate at 12 months follow-up) in 48.2% (95% CI, 44.0–52.3). Study arms with PVI only achieved the primary outcome in 55.8% (95% CI, 50.0%–61.7%), while for PVI + additional lesion sets this was 44.1% (95% CI, 38.7%–49.4%).

### Outcomes Over Time

**Figure 1.**
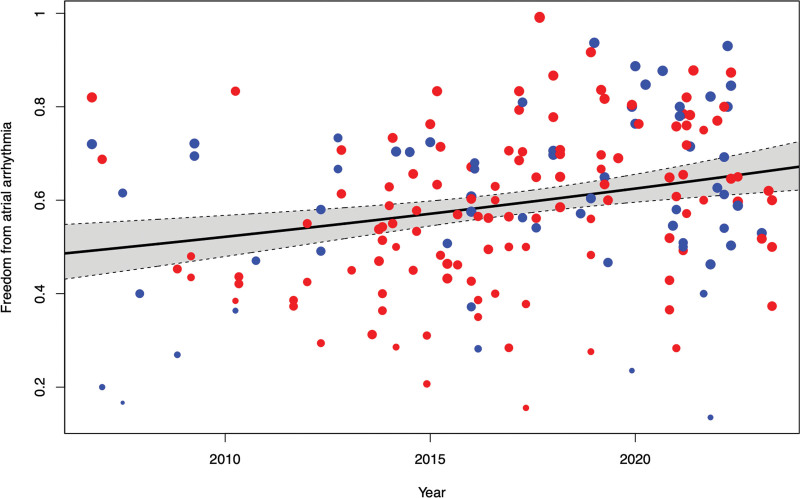
**Meta-regression shows improvements in freedom from atrial arrhythmia over time.** Each marker represents a study arm, with size being proportional to study size. Blue indicates pulmonary vein isolation (PVI) only, while red indicates PVI plus additional lesions. A 1-year increase in publication year was associated with a 1.8% better outcome (CI, 0.8%–2.8%; *P*<0.028).

Figure 1 displays each study arm as a data point, showing freedom from atrial arrhythmia at 12 months against time. A meta-regression was performed to show the gradual increase in efficacy over time. Additional lesions sets as a whole were associated with a nonsignificant trend toward worse outcomes (−5.3% change in freedom from atrial arrhythmia 95% CI, −13.8% to 3.1%; *P*=0.26). A 1-year increase in publication year was associated with a 1.8% better outcome (CI, 0.8%–2.8%; *P*<0.028).

### Procedural Factors Over Time

**Figure 2.**
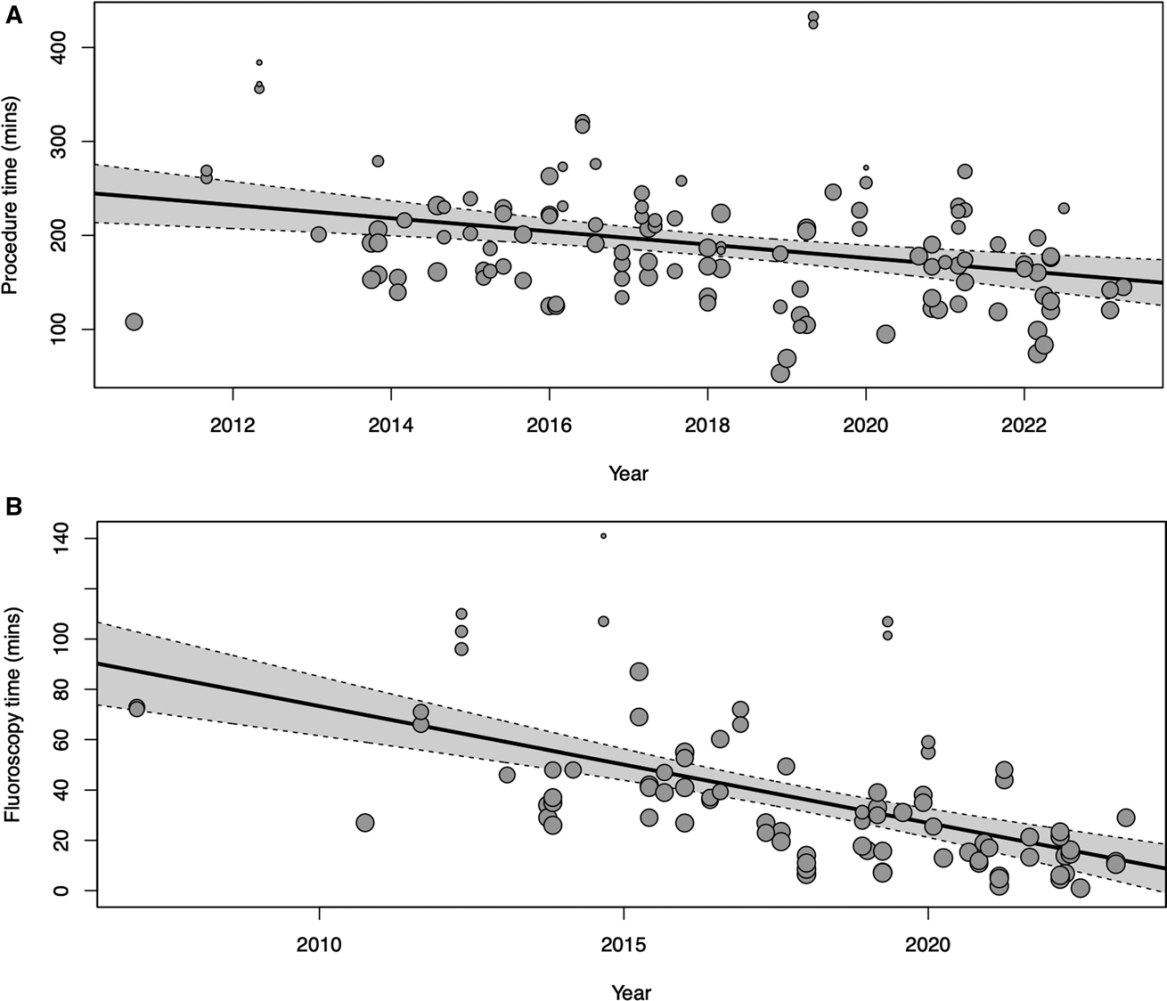
**Meta-regression shows reductions in procedure and fluoroscopy time by study year. A**, Procedure, and (**B**) fluoroscopy. Each marker represents a study arm, with size being proportional to study size. Procedure time has reduced by 7.0 minutes per year (CI, −10.8 to – 3.3; *P*<0.001), while fluoroscopy time has reduced by 4.6 minutes per year (CI, −6.0 to −3.3; *P*<0.0001).

Figure 2 shows the significant reduction in procedure time (a 7.0 min/year [CI, −10.8 to – 3.3]; *P*<0.001) and fluoroscopy time (a 4.6 min/year [CI, −6.0 to −3.3]; *P*<0.0001). There was no significant change in major complication rates over time (Figure S3; 0.021% change/year [95% CI, −0.77% to 0.44%]; *P*=0.44). Secondary analyses using restricted cubic splines (3 and 4 knots) also did not show any significant relationship between year and complication rate.

### Effect of Lesion Set on Freedom From Atrial Arrhythmia

**Figure 3.**
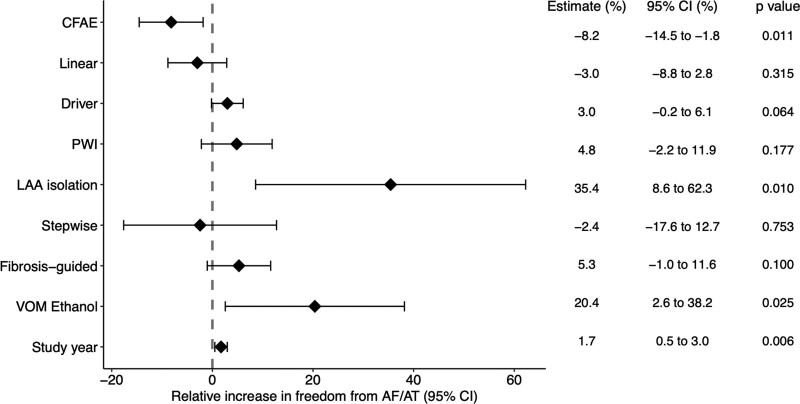
**The effect of predictors on freedom from atrial arrhythmia at 12 months.** AF indicates atrial fibrillation; AT, atrial tachycardia; CFAE, complex fractionated atrial electrogram; LAA, left atrial appendage; PWI, posterior wall isolation; and VOM, vein of Marshall.

A summary of these results is shown in Figure 3. Study year was included in this analysis to adjust for the effect of study year on outcomes. Left atrial appendage isolation (2 study arms) and vein of Marshall ethanol ablation (1 study arm) was associated with improved outcomes. CFAE ablation (47 study arms) was associated with worse outcomes. PWI (25 study arms), linear ablation (56 study arms), driver ablation (13 study arms), a stepwise strategy (6 study arms), and fibrosis-guided ablation (5 study arms) did not have a significant effect on outcomes. After adjusting for ablation strategy, study year maintained a significant positive associated with outcome. Secondary analysis on RCTs only showed driver ablation had a positive effect on reducing atrial arrhythmia recurrence, and the other results were similar (Figure S4).

### Cumulative Meta-Analysis

**Figure 4.**
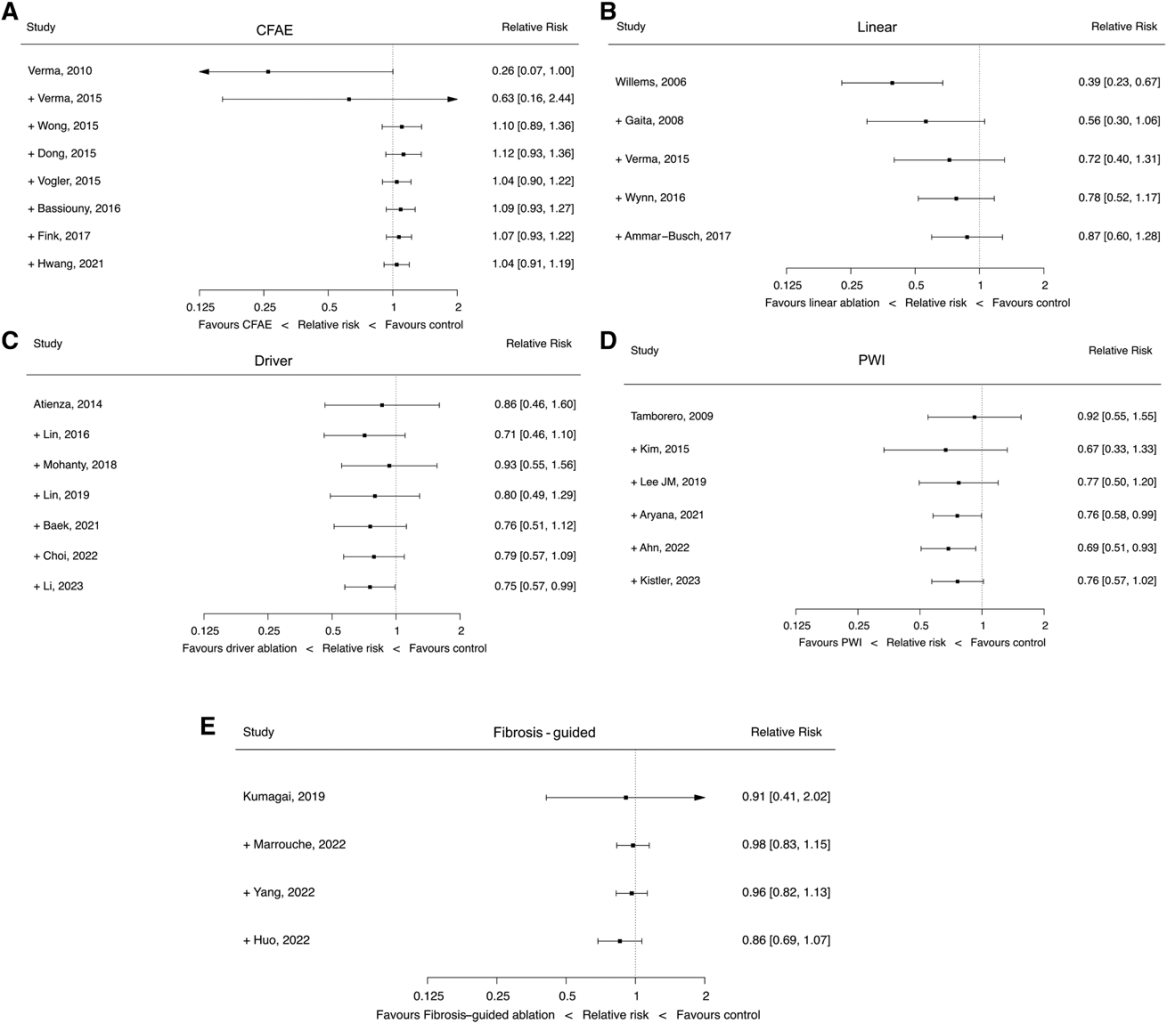
**Cumulative meta-analyses of additional lesion sets in persistent atrial fibrillation (PsAF) ablation.** Studies are arranged and cumulated chronologically based on publication year. Complex fractionated atrial electrogram (CFAE) ablation (**A**), linear ablation (**B**), driver ablation (**C**), posterior wall isolation ([PWI], **D**), and fibrosis-guided ablation (**E**).

To assess the evolution over time of each major adjunctive ablation strategy, we performed cumulative meta-analysis. Here, we consider only RCTs. Only studies with at least PVI in the control arm were included. The intervention arms were adjunctive ablation strategies. While CFAE ablation showed initial promise, after 3 RCTs, the point estimate appeared firmly neutral with a trend toward harm (Figure 4A). A similar pattern can be seen for linear ablation, with initial positive results, but subsequently shown to be neutral (Figure 4B). Driver ablation, after the most recent trial, appears to show evidence of benefit (Figure 4C). PWI showed a statistically significant positive effect after 5 RCTs but is now nonsignificant after the recent CAPLA trial (Figure 4D). Fibrosis-guided ablation does not have a significant effect (Figure 4E). There was evidence of heterogeneity of effect between trials of driver ablation, PWI, and linear ablation (Table S1).

### Trial Sequential Analysis

The cumulative meta-analyses raise an important question: How do we know if a novel therapy has had sufficient evaluation to be definitively deemed efficacious, harmful, or neutral. As shown by the cumulative meta-analysis results in Figure 4, initially promising therapies may subsequently be found to be ineffective. Conversely, with small numbers of patients in individual studies, it may take several studies before a definitive benefit is shown. However, it is not clear from conventional meta-analysis whether a definitive answer has been reached, resulting in repeated studies being conducted to answer the same question, wasting significant time and resources conducting trials for therapies that have already been conclusively shown to be ineffective, where further study is futile. TSA aims to address these issues.^13^

**Figure 5.**
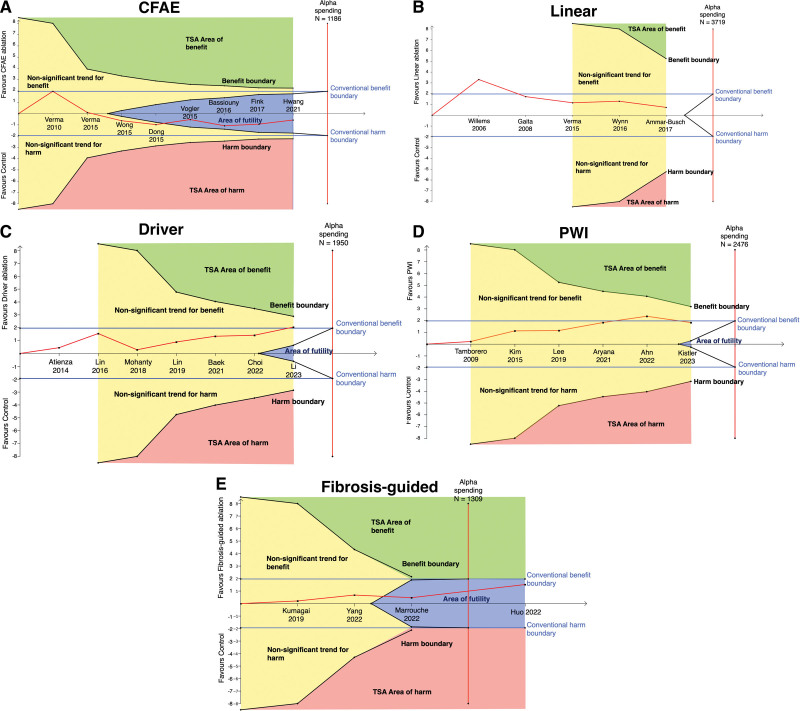
**Trial sequential analyses (TSA) of additional lesion sets in persistent atrial fibrillation (PsAF) ablation.** Studies are arranged and cumulated chronologically based on publication year. Complex fractionated atrial electrogram (CFAE) ablation (**A**), linear ablation (**B**), driver ablation (**C**), posterior wall isolation ([PWI], **D**), and (**E**) fibrosis-guided ablation.

For the reader unfamiliar with TSA, some explanation is required to interpret the following Figure 5.^21^ Each graph is divided into 4 zones, TSA area of benefit (green), TSA area of harm (red), area of futility (blue), and nonstatistically significant zone (yellow). The conventional boundaries (blue lines) indicate where conclusions of benefit or harm would be reached with traditional meta-analysis, which do not consider if the total cumulative information size (ie, number of patients) is adequate to reach a conclusion. TSA, however, uses adequate information size to understand if the current data are sufficient to make a conclusion of benefit, harm, or futility. The nonsignificant zones indicate that further study is required. While the area of futility suggests sufficient data have been collected to conclude that further research is unlikely to change the outcome into harm or benefit, it is important to note that TSA is influenced by the definition of adequate information size, which is derived from the estimate of treatment effect (that was pragmatically set to a relative risk reduction of 20% in this analysis).

TSA shows further study of CFAE ablation is futile (Figure 5A). The last 3 RCTs were in fact were not required to reach this conclusion, with TSA showing that sufficient information had been gathered following the results of the study by Vogler et al.^22^ Although showing initial promise, linear ablation now does not seem to significantly impact outcomes but has not yet had sufficient participants studied to be definitively deemed futile (Figure 5B). The addition of the latest trial has meant driver ablation crosses the conventional meta-analysis threshold for significant benefit; however, TSA sufficient power has not been reached to make this conclusion definite (Figure 5C). PWI has no significant effect on freedom from AF in both traditional meta-analysis and using TSA; however, futility has not been reached. (Figure 5D). Fibrosis-guided ablation does not have evidence of benefit and TSA concludes it would be futile to study this further (Figure 5E).

Additional analyses with an estimate of treatment effect at 15% relative risk reduction broadly supported the same conclusions (Figure S5).

## DISCUSSION

To our knowledge, we present the first meta-analysis and meta-regression-based description of the temporal evolution of ablation for PsAF. We demonstrate that there has been a modest and sustained improvement in success rates for ablation of PsAF over time, although this does not seem to be due to adjunctive ablation as a whole. Through cumulative meta-analysis and TSA, we show that several adjunctive ablation strategies that initially appeared positive were subsequently shown to be ineffective with more randomized data. We confirm that CFAE ablation is ineffective and further study of this treatment would be futile. We show that PWI, while having recently appeared promising, does not have sufficient data to support its efficacy and should not be performed routinely outside of research settings.

### Modest but Sustained Increased in PsAF Ablation Success Rates Over Time

We show ablation outcomes from PVI, the cornerstone of PsAF ablation, has improved modestly year on year. This is most likely due to improved PVI durability due to technological advances, including contact force, irrigation, 3D mapping, single shot technologies, and accumulating clinical experience.^6^ Similarly, procedure and fluoroscopy times have reduced markedly over this time period. This is also likely due to a combination of technological advances (in particular 3D mapping for fluoroscopy times and 1 shot/balloon technologies for procedure times) and procedural experience.

Complication rates do not seem to have changed over time. This may be due to higher risk patient populations having AF ablation in recent years, in particularly the heart failure population following evidence of prognostic benefit from RCTs.^23^ Other possibilities include the shift from few high-volume centers to more lower volume AF ablation centers, who may have higher complication rates.^24,25^ Additionally, given the significant variability in how studies report complications, it is possible that any change in complication incidence is masked by this limitation.

### CFAE Ablation Does Not Improve Outcomes in PsAF Ablation, and May Worsen Them

Over the years, many additional lesion sets, beyond PVI, have been evaluated but none was universally accepted as a standard of care. Our meta-regression shows that CFAE ablation may in fact be harmful, possibility due to increased recurrences due to AT.^26^ This is in line with our previous analysis and other studies.^8,10,27^

In a novel analysis using TSA, we conclude that further study into CFAE ablation should be abandoned. In fact, the same conclusion would have been reached in 2015, even without the last 3 trials. Research resources and efforts would, therefore, be better redirected to alternative approaches and clinical problems.

### Insufficient Evidence to Support PWI in First-Time PsAF Ablation

PWI has been seen as a promising adjunctive ablation strategy in recent years.^28^ Promising early data combined with mechanistic plausibility has led to much enthusiasm about this strategy.^29–31^ In our study, in line with previous meta-analysis,^28^ the cumulative meta-analysis demonstrated a benefit of PWI in PsAF outcomes, up until the recent CAPLA study. Without further analysis, it may have been tempting to conclude that PWI should, therefore, be performed routinely in PsAF ablation. However, through the use of TSA we conclude, before and after CAPLA, that further studies are needed. This conclusion is supported by Kim et al.^32^ Although they studied redo procedures only, and therefore the data were not included in the present study, the finding that PWI provided no benefit reaffirms the importance of adequate data before firm conclusions can be made. Finally, our meta-regression, with its larger dataset but with the limitations of inclusion of observational data, also found no effect of PWI on rhythm outcomes. Our analysis shows that PWI should not be performed routinely in first-time PsAF ablation outside of research settings. PWI using pulsed-field ablation in particular requires evaluation given the potentially favorable safety profile and potential for more durable lesions.^33^

### The Importance of Adequately Powered RCTs Before Adoption of Novel Therapies

Through the use of cumulative meta-analyses, we have shown how some adjunctive ablation strategies may initially seem promising, but after several RCTs, may be found to be ineffective. This is often the case for many novel interventional therapies, within and outside of cardiac electrophysiology, and may partly reflect publication bias of small single-center studies that report positive results of novel treatments. From our analysis, this was particularly evident for linear ablation, which appeared to have a positive effect on outcomes in the initial trial, but subsequently appeared to have a neutral effect. We have shown how TSA may be helpful to prevent premature conclusions of efficacy being reached.

### Areas Requiring Further Study

ERASE AF was an RCT of PVI versus PVI + substrate ablation guided by low-voltage areas.^34^ The primary outcome in this study showed an impressive 38% reduction in atrial arrhythmia recurrence at 1 year in the substrate ablation arm. This finding is somewhat surprising given that only one-third of the substrate arm had low-voltage areas, and therefore two-third of this arm had PVI alone. Additionally, in the patients of the study that did not have low-voltage areas (206/324 patients, 64%) who therefore received the same treatment in both arms (PVI alone), there was still a (nonsignificant) reduction in the primary outcome of 34%. It is difficult to explain why there should be such a difference between the 2 groups who should have received identical treatment. These unusual findings in combination with the findings of our study suggest further research into low-voltage area-guided substrate ablation is needed before clinical use. Although our TSA concludes further study would be futile, there are significant differences between the methodologies of the studies (including MRI guided versus electroanatomical guided), which may mean treating them as one technique is not appropriate.

Our analyses show that left atrial appendage isolation may have promise; however, this was based only on 2 studies and is heavily based on the BELIEF trial.^35^ Similarly vein of Marshall ethanol ablation is based on only the VENUS trial.^36^ Therefore, further RCTs are important before any conclusions can be made. Safety end points for left atrial appendage isolation are of particular importance given potential concerns regarding cerebral thromboembolism.^37^

Driver ablation represents a heterogenous group of procedural strategies, and as a group, meta-analysis suggests overall benefit. Our TSA suggests, however, that no conclusion can be made currently, and further study is needed. Additionally, given the heterogeneity in strategies, driver ablation may not be ideally suited to pooled effect estimates with meta-analysis.

In the studies that we included in our analysis, the adjunctive ablations were performed in all patients allocated to those treatment groups. With the increasing recognition of the existence of different phenotypes of AF, sustained by different AF mechanisms,^38,39^ it is possible that these adjunctive ablation strategies can be effective if tailored and individualized to each patient’s underlying AF mechanism.

### Limitations

The meta-regression analysis used in this study pools data from multiple study arms for observations on associations. These observations are therefore not based on randomized comparisons and are vulnerable to confounding. Additionally, data within the trials were not sufficiently granular or detailed enough to separate out the causes for the associations seen. For example, we could not determine the cause for the increased success rates in PsAF ablation over time. There was evidence of heterogeneity between trials of linear and driver ablation, which may affect the validity of the meta-analyses for these lesion sets. The observed heterogeneity is not surprising given the differences in protocols and ablation strategies between studies. This highlights an important limitation of meta-analyses for catheter ablation as compared with medical treatment meta-analyses. While in drug trials, different studies are generally comparable, ablation trials are often very heterogenous with regards to ablation methodology, making comparison between trials challenging. Similar to power calculation, TSA requires specification of the estimate of treatment effect. This estimated treatment effect can have a significant impact on the results of the analysis. A Cochrane expert panel preferred to interpret meta-analysis based on estimated intervention effect and uncertainty rather than rely on the binary interpretations provided by TSA.^40^ To help mitigate the impact of a single estimate, we have presented results at another estimate of treatment effect size in the Supplemental Material. Different monitoring methods (eg, 24-hour rhythm monitoring versus implantable loop recorder) in different trials may have a significant impact on reported freedom from AF, and this may affect the results of the pooled effects reported in our study. Effect sizes of strategies with limited numbers of studies (such as left atrial appendage or vein of Marshall ethanol ablation) may be significantly over or underestimated and require further study.

### Conclusions

Overall success rates for catheter ablation of PsAF have increased steadily over time, with a reduction in procedure/fluoroscopy times. No adjunctive lesion set, in addition to PVI, has been conclusively demonstrated to be beneficial. Through the use of cumulative meta-analyses and TSA, we show that some ablation strategies may initially seem promising, but after several RCTs, may be found to be ineffective, highlighting the importance of adequately powered RCTs before widespread adoption of novel therapies. This is particularly apparent for PWI, which despite recent promise, does not currently have sufficient evidence to support its routine adoption, and warrants further study.

## ARTICLE INFORMATION

### Sources of Funding

ASau is funded by a British Heart Foundation (BHF) clinical research training fellowship (FS/CRTF/21/24183) and was supported by a National Institute for Health Research (NIHR) Academic Clinical Fellowship (ACF-2019-21-001). Drs Ng and Peters are supported by the BHF (RG/F/22/110078 and RE/18/4/34215). Dr Ng is also supported by the NIHR Imperial Biomedical Research Center. Dr Howard is supported by the BHF (FS/ICRF/22/26039).

### Disclosures

None.

### Supplemental Material

Table S1

Figures S1–S6

## Supplementary Material


