# Supplementary material for: Temporal Trends and Lesion Sets for Persistent Atrial Fibrillation Ablation: A Meta-Analysis With Trial Sequential Analysis and Meta-Regression
Source: Circ Arrhythm Electrophysiol. 2023 Aug 17;16(9):e011861. doi: 10.1161/CIRCEP.123.011861 (PMC10510845; doi:10.1161/CIRCEP.123.011861)
Supplement: Supplementary file 1 [file hae-16-e011861-s001.pdf]

SUPPLEMENTAL MATERIAL

Supplemental Table I
Random effects meta-analysis measures of heterogeneity

Meta-analysis	Q	df	P value for heterogeneity	I^2
CFAE ablation	11.26	7	0.13	4.36%
Linear ablation	21.09	4	0.0003	85.95%
Driver ablation	14.37	6	0.026	58.75%
Posterior wall isolation	11.55	5	0.04	55.86%
Fibrosis-guided ablation	4.16	3	0.24	40.61%

Supplemental Figure I
Sources of identified studies

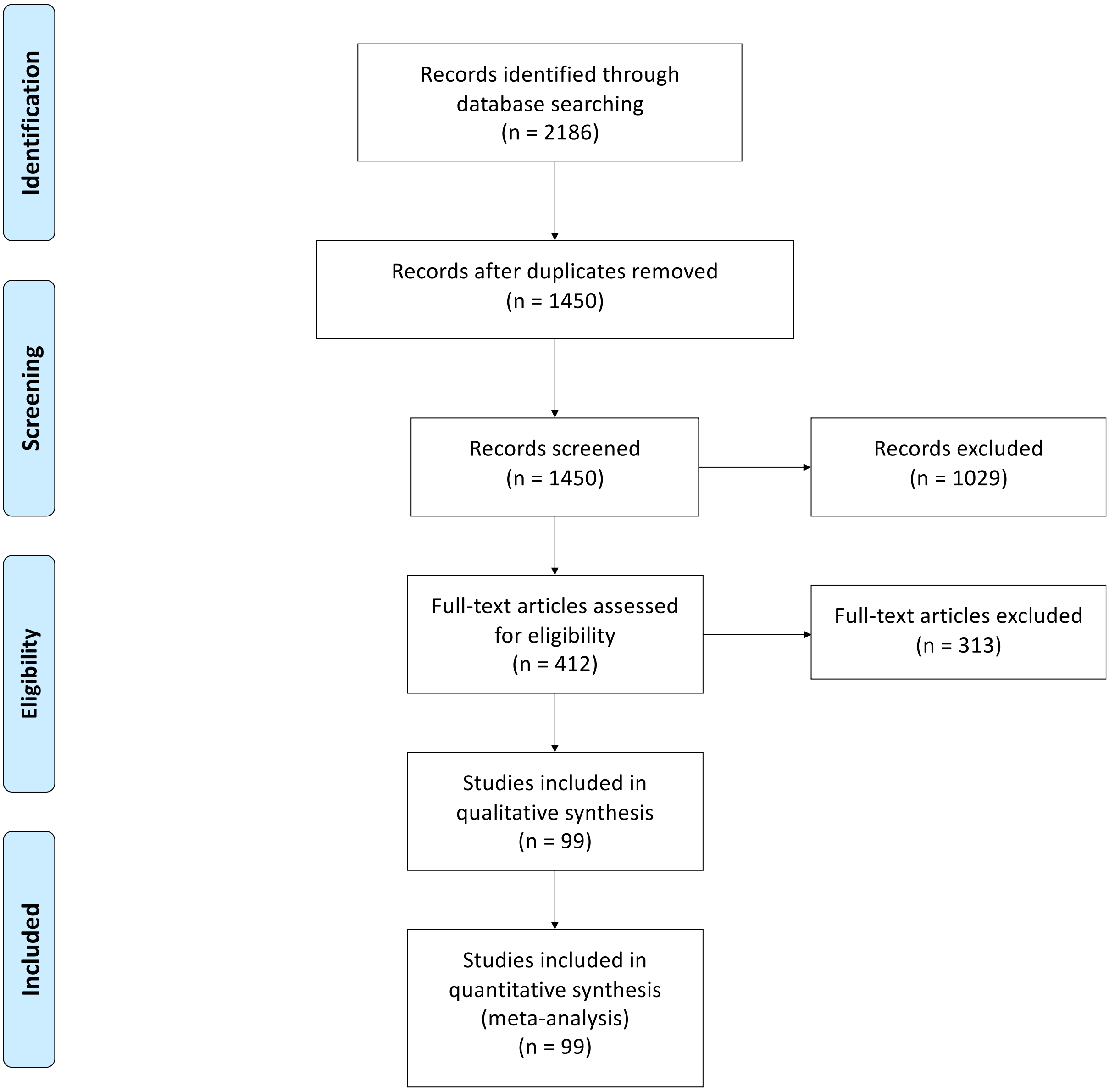

Supplemental Figure II

Funnel plot to assess for publication bias

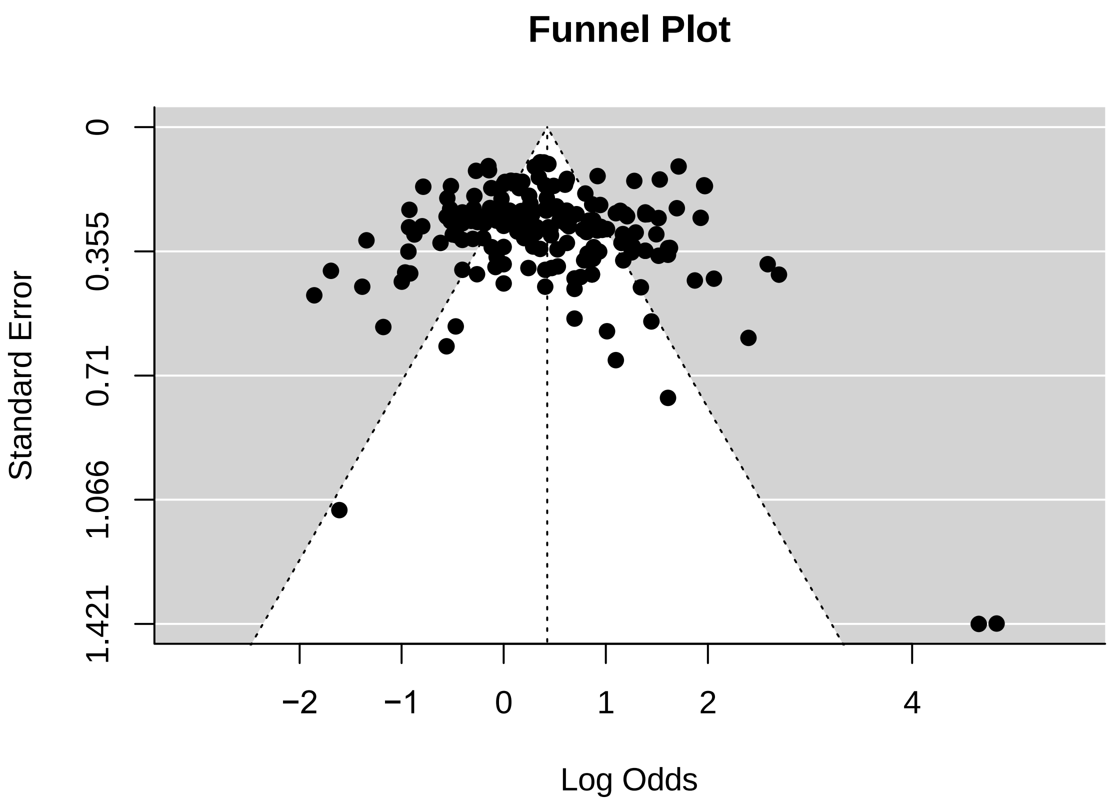

Supplemental Figure III

Meta-regression shows no change in complication rates over time. Each marker represents a study arm, with size being proportional to study size.

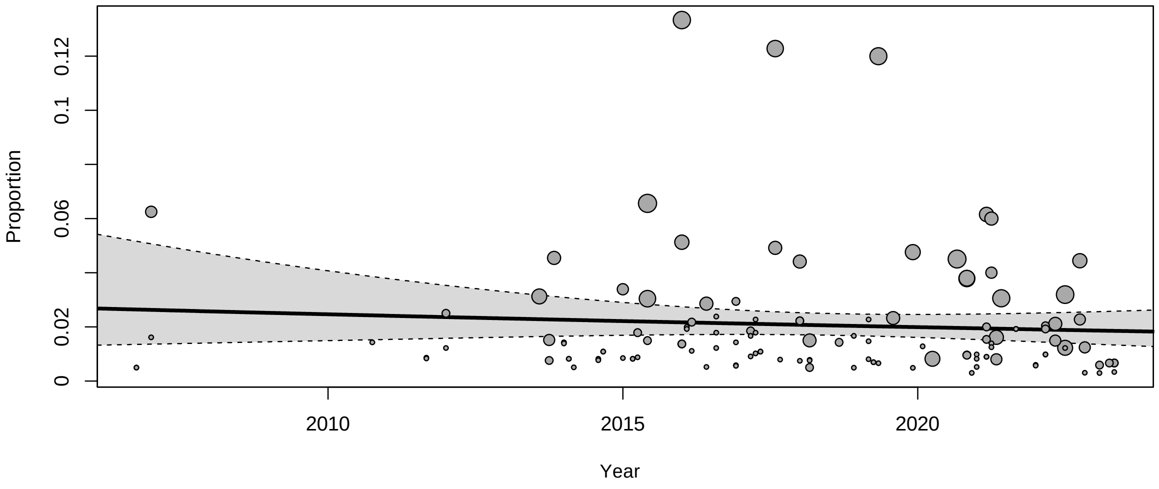

Supplemental Figure IV

Meta-regression secondary analysis including RCTs only**.** The effect of predictors on freedom from atrial arrhythmia at 12 months. AF, atrial fibrillation; AT, atrial tachycardia; CFAE, complex fractionated atrial electrogram; CI, confidence interval; PWI, posterior wall isolation; LAA, left atrial appendage; VOM, vein of Marshall.

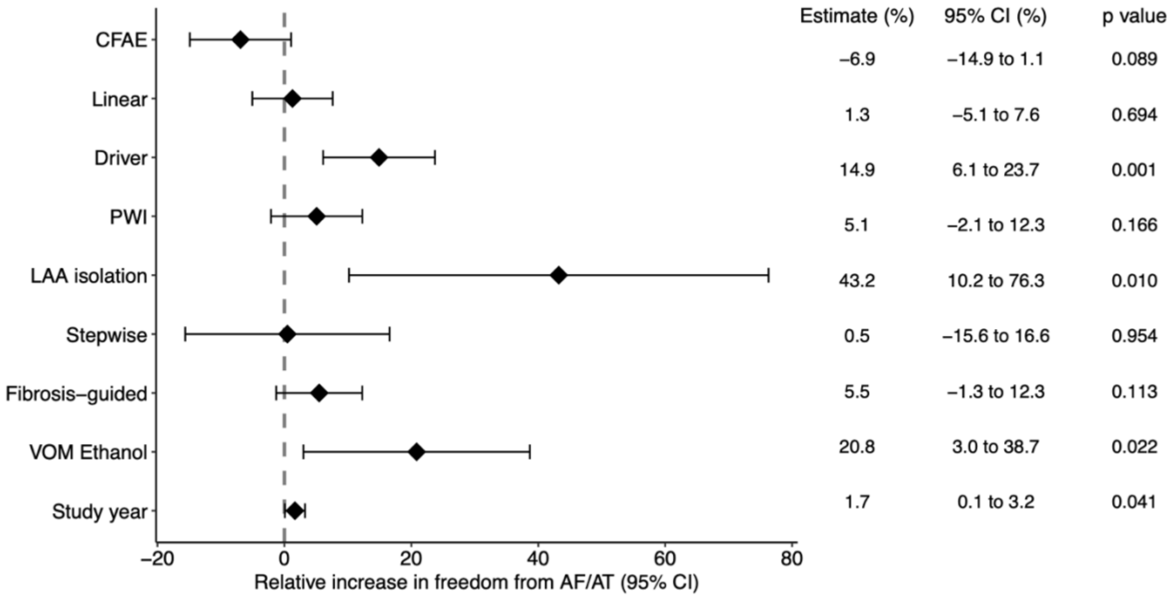

Supplemental Figure V

Trial sequential analyses (TSA) of additional lesion sets in PsAF ablation. Sensitivity analysis with estimate of treatment effect set at 15%.
CFAE ablation (A), linear ablation (B), and driver ablation (C), PWI (D), Fibrosis-guided ablation (E).
CFAE, complex fractionated atrial electrogram; PWI, posterior wall isolation

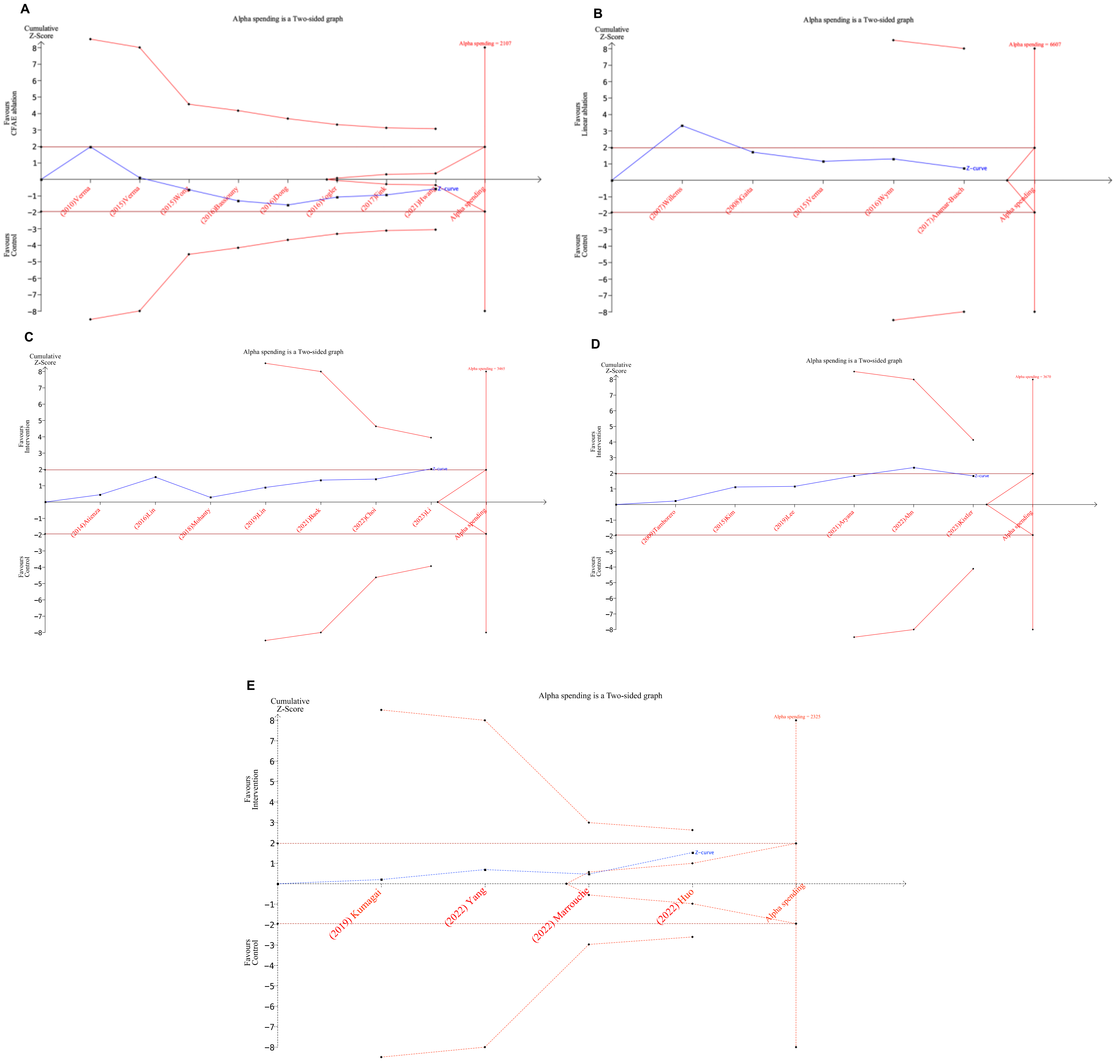

Supplemental Figure VI

Risk of bias graph

Study ID	D1	D2	D3	D4	D5	Overall
Tamborero 2009	!	!	+	+	!	!
Kim 2015	!	!	+	!	!	!
Lee 2019	!	!	+	!	!	!
Aryana 2021	!	!	+	+	!	!
Ahn 2022	!	-	+	!	!	-
Verma 2010	+	-	+	!	!	-
Verma 2015	+	!	+	+	+	!
Wong 2015	!	!	+	!	!	!
Dong 2015	!	!	+	+	!	!
Vogler 2015	!	!	+	!	!	!
Bassiouny 2016	!	!	+	!	!	!
Fink 2017	!	!	+	!	!	!
Hwang 2021	-	!	+	!	!	-
Willems 2006	!	!	+	!	!	!
Gaita 2008	!	!	+	!	!	!
Wynn 2016	!	!	+	!	!	!
Ammar-Busch 2017	!	!	+	!	!	!
Atienza 2014	!	!	+	+	!	!
Lin 2016	!	!	+	!	!	!
Mohanty 2018	!	+	+	!	!	!
Lin 2019	!	!	+	!	!	!
Baek 2021	!	-	+	+	!	-
Choi 2023	+	!	+	-	!	-
Kistler 2023	!	!	+	+	!	!
Li 2023	!	+	+	-	!	-
Marrouche 2022	+	+	+	+	!	!
Yang 2022	+	!	+	+	!	!
Huo 2022	+	!	+	+	+	!

+ Low risk
! Some concerns
- High risk

D1 Randomisation process
D2 Deviations from the intended interventions
D3 Missing outcome data
D4 Measurement of the outcome
D5 Selection of the reported result

Full list of included studies

1. Kojodjojo P, O'Neill MD, Lim PB, Malcolm-Lawes L, Whinnett ZI, Salukhe TV, et al. Pulmonary venous isolation by antral ablation with a large cryoballoon for treatment of paroxysmal and persistent atrial fibrillation: medium-term outcomes and non-randomised comparison with pulmonary venous isolation by radiofrequency ablation. Heart. 2010;96(17):1379-84.
2. Ahn J, Shin DG, Han SJ, Lim HE. Does isolation of the left atrial posterior wall using cryoballoon ablation improve clinical outcomes in patients with persistent atrial fibrillation? A prospective randomized controlled trial. Europace. 2022;24(7):1093-101.
3. Aksu T, Guler TE, Bozyel S, Yalin K, Lakkireddy D, Gopinathannair R. Initial experience with fractionation mapping-guided ablation strategy in patients with long-standing persistent atrial fibrillation. J Interv Card Electrophysiol. 2021;61(2):405-13.
4. Ammar-Busch S, Bourier F, Reents T, Semmler V, Telishevska M, Kathan S, et al. Ablation of Complex Fractionated Electrograms With or Without ADditional LINEar Lesions for Persistent Atrial Fibrillation (The ADLINE Trial). J Cardiovasc Electrophysiol. 2017;28(6):636-41.
5. Andrade JG, Yao RRJ, Deyell MW, Hawkins NM, Rizkallah J, Jolly U, et al. Clinical assessment of AF pattern is poorly correlated with AF burden and post ablation outcomes: A CIRCA-DOSE sub-study. J Electrocardiol. 2020;60:159-64.
6. Aryana A, Allen SL, Pujara DK, Bowers MR, O'Neill PG, Yamauchi Y, et al. Concomitant Pulmonary Vein and Posterior Wall Isolation Using Cryoballoon With Adjunct Radiofrequency in Persistent Atrial Fibrillation. JACC Clin Electrophysiol. 2021;7(2):187-96.
7. Atienza F, Almendral J, Ormaetxe JM, Moya A, Martínez-Alday JD, Hernández-Madrid A, et al. Comparison of radiofrequency catheter ablation of drivers and circumferential pulmonary vein isolation in atrial fibrillation: a noninferiority randomized multicenter RADAR-AF trial. J Am Coll Cardiol. 2014;64(23):2455-67.
8. Baek YS, Kwon OS, Lim B, Yang SY, Park JW, Yu HT, et al. Clinical Outcomes of Computational Virtual Mapping-Guided Catheter Ablation in Patients With Persistent Atrial Fibrillation: A Multicenter Prospective Randomized Clinical Trial. Front Cardiovasc Med. 2021;8:772665.
9. Bassiouny M, Saliba W, Hussein A, Rickard J, Diab M, Aman W, et al. Randomized Study of Persistent Atrial Fibrillation Ablation: Ablate in Sinus Rhythm Versus Ablate Complex-Fractionated Atrial Electrograms in Atrial Fibrillation. Circ Arrhythm Electrophysiol. 2016;9(2):e003596.
10. Bertaglia E, Senatore G, De Michieli L, De Simone A, Amellone C, Ferretto S, et al. Twelve-year follow-up of catheter ablation for atrial fibrillation: A prospective, multicenter, randomized study. Heart Rhythm. 2017;14(4):486-92.
11. Boveda S, Metzner A, Nguyen DQ, Chun KRJ, Goehl K, Noelker G, et al. Single-Procedure Outcomes and Quality-of-Life Improvement 12 Months Post-Cryoballoon Ablation in Persistent Atrial Fibrillation: Results From the Multicenter CRYO4PERSISTENT AF Trial. JACC Clin Electrophysiol. 2018;4(11):1440-7.
12. Caponi D, Corleto A, Scaglione M, Blandino A, Biasco L, Cristoforetti Y, et al. Ablation of atrial fibrillation: does the addition of three-dimensional magnetic resonance imaging of the left atrium to electroanatomic mapping improve the clinical outcome?: a randomized comparison of Carto-Merge vs. Carto-XP three-

dimensional mapping ablation in patients with paroxysmal and persistent atrial fibrillation. Europace. 2010;12(8):1098-104.
13. Choudry S, Mansour M, Sundaram S, Nguyen DT, Dukkipati SR, Whang W, et al. RADAR: A Multicenter Food and Drug Administration Investigational Device Exemption Clinical Trial of Persistent Atrial Fibrillation. Circ Arrhythm Electrophysiol. 2020;13(1):e007825.
14. Chun JKR, Bordignon S, Last J, Mayer L, Tohoku S, Zanchi S, et al. Cryoballoon Versus Laserballoon: Insights From the First Prospective Randomized Balloon Trial in Catheter Ablation of Atrial Fibrillation. Circ Arrhythm Electrophysiol. 2021;14(2):e009294.
15. Combes S, Jacob S, Combes N, Karam N, Chaumeil A, Guy-Moyat B, et al. Predicting favourable outcomes in the setting of radiofrequency catheter ablation of long-standing persistent atrial fibrillation: a pilot study assessing the value of left atrial appendage peak flow velocity. Arch Cardiovasc Dis. 2013;106(1):36-43.
16. Conti S, Weerasooriya R, Novak P, Champagne J, Lim HE, Macle L, et al. Contact force sensing for ablation of persistent atrial fibrillation: A randomized, multicenter trial. Heart Rhythm. 2018;15(2):201-8.
17. Davies EJ, Clayton B, Lines I, Haywood GA. Persistent Atrial Fibrillation Ablation using the Tip-Versatile Ablation Catheter. Heart Lung Circ. 2016;25(7):645-51.
18. De Bortoli A, Ohm OJ, Hoff PI, Sun LZ, Schuster P, Solheim E, et al. Long-term outcomes of adjunctive complex fractionated electrogram ablation to pulmonary vein isolation as treatment for non-paroxysmal atrial fibrillation. J Interv Card Electrophysiol. 2013;38(1):19-26.
19. De Greef Y, Buysschaert I, Schwagten B, Stockman D, Tavernier R, Duytschaever M. Duty-cycled multi-electrode radiofrequency vs. conventional irrigated point-by-point radiofrequency ablation for recurrent atrial fibrillation: comparative 3-year data. Europace. 2014;16(6):820-5.
20. DeLurgio DB, Crossen KJ, Gill J, Blauth C, Oza SR, Magnano AR, et al. Hybrid Convergent Procedure for the Treatment of Persistent and Long-Standing Persistent Atrial Fibrillation: Results of CONVERGE Clinical Trial. Circ Arrhythm Electrophysiol. 2020;13(12):e009288.
21. Di Biase L, Burkhardt JD, Mohanty P, Mohanty S, Sanchez JE, Trivedi C, et al. Left Atrial Appendage Isolation in Patients With Longstanding Persistent AF Undergoing Catheter Ablation: BELIEF Trial. J Am Coll Cardiol. 2016;68(18):1929-40.
22. Dixit S, Marchlinski FE, Lin D, Callans DJ, Bala R, Riley MP, et al. Randomized ablation strategies for the treatment of persistent atrial fibrillation: RASTA study. Circ Arrhythm Electrophysiol. 2012;5(2):287-94.
23. Dong JZ, Sang CH, Yu RH, Long DY, Tang RB, Jiang CX, et al. Prospective randomized comparison between a fixed '2C3L' approach vs. stepwise approach for catheter ablation of persistent atrial fibrillation. Europace. 2015;17(12):1798-806.
24. Du X, Guo L, He X, Jia Y, Wu J, Long D, et al. A comparison of the real world effectiveness of catheter ablation and drug therapy in atrial fibrillation patients in a Chinese setting. BMC Cardiovasc Disord. 2017;17(1):204.
25. Estner HL, Hessling G, Biegler R, Schreieck J, Fichtner S, Wu J, et al. Complex fractionated atrial electrogram or linear ablation in patients with persistent atrial fibrillation--a prospective randomized study. Pacing Clin Electrophysiol. 2011;34(8):939-48.

26. Fiala M, Wichterle D, Bulková V, Sknouril L, Nevralová R, Toman O, et al. A prospective evaluation of haemodynamics, functional status, and quality of life after radiofrequency catheter ablation of long-standing persistent atrial fibrillation. Europace. 2014;16(1):15-25.
27. Fink T, Schlüter M, Heeger CH, Lemes C, Maurer T, Reissmann B, et al. Stand-Alone Pulmonary Vein Isolation Versus Pulmonary Vein Isolation With Additional Substrate Modification as Index Ablation Procedures in Patients With Persistent and Long-Standing Persistent Atrial Fibrillation: The Randomized Alster-Lost-AF Trial (Ablation at St. Georg Hospital for Long-Standing Persistent Atrial Fibrillation). Circ Arrhythm Electrophysiol. 2017;10(7).
28. Gaita F, Caponi D, Scaglione M, Montefusco A, Corleto A, Di Monte F, et al. Long-term clinical results of 2 different ablation strategies in patients with paroxysmal and persistent atrial fibrillation. Circ Arrhythm Electrophysiol. 2008;1(4):269-75.
29. Gallagher MM, Yi G, Gonna H, Leung LWM, Harding I, Evranos B, et al. Multi-catheter cryotherapy compared with radiofrequency ablation in long-standing persistent atrial fibrillation: a randomized clinical trial. Europace. 2021;23(3):370-9.
30. Haldar S, Khan HR, Boyalla V, Kralj-Hans I, Jones S, Lord J, et al. Catheter ablation vs. thoracoscopic surgical ablation in long-standing persistent atrial fibrillation: CASA-AF randomized controlled trial. Eur Heart J. 2020;41(47):4471-80.
31. Han SW, Shin SY, Im SI, Na JO, Choi CU, Kim SH, et al. Does the amount of atrial mass reduction improve clinical outcomes after radiofrequency catheter ablation for long-standing persistent atrial fibrillation? Comparison between linear ablation and defragmentation. Int J Cardiol. 2014;171(1):37-43.
32. Honarbakhsh S, Dhillon G, Abbass H, Waddingham PH, Dennis A, Ahluwalia N, et al. Noninvasive electrocardiographic imaging-guided targeting of drivers of persistent atrial fibrillation: The TARGET-AF1 trial. Heart Rhythm. 2022;19(6):875-84.
33. Honarbakhsh S, Schilling RJ, Providencia R, Dhillon G, Bajomo O, Keating E, et al. Ablation guided by STAR-mapping in addition to pulmonary vein isolation is superior to pulmonary vein isolation alone or in combination with CFAE/linear ablation for persistent AF. J Cardiovasc Electrophysiol. 2021;32(2):200-9.
34. Huang X, Chen Y, Huang Y, Zhao H, Chen Y, He L, et al. Clinical efficacy of irrigated catheter application of amiodarone during ablation of persistent atrial fibrillation. Clin Cardiol. 2017;40(12):1333-8.
35. Hwang J, Park HS, Han S, Lee CH, Kim IC, Cho YK, et al. Ablation of persistent atrial fibrillation based on high density voltage mapping and complex fractionated atrial electrograms: A randomized controlled trial. Medicine (Baltimore). 2021;100(31):e26702.
36. Inoue K, Hikoso S, Masuda M, Furukawa Y, Hirata A, Egami Y, et al. Pulmonary vein isolation alone vs. more extensive ablation with defragmentation and linear ablation of persistent atrial fibrillation: the EARNEST-PVI trial. Europace. 2021;23(4):565-74.
37. Kim IS, Lim B, Shim J, Hwang M, Yu HT, Kim TH, et al. Clinical Usefulness of Computational Modeling-Guided Persistent Atrial Fibrillation Ablation: Updated Outcome of Multicenter Randomized Study. Front Physiol. 2019;10:1512.
38. Kim JS, Shin SY, Na JO, Choi CU, Kim SH, Kim JW, et al. Does isolation of the left atrial posterior wall improve clinical outcomes after radiofrequency catheter ablation for persistent atrial fibrillation?: A prospective randomized clinical trial. Int J Cardiol. 2015;181:277-83.

39. Kim TH, Uhm JS, Kim JY, Joung B, Lee MH, Pak HN. Does Additional Electrogram-Guided Ablation After Linear Ablation Reduce Recurrence After Catheter Ablation for Longstanding Persistent Atrial Fibrillation? A Prospective Randomized Study. J Am Heart Assoc. 2017;6(2).
40. Kircher S, Arya A, Altmann D, Rolf S, Bollmann A, Sommer P, et al. Individually tailored vs. standardized substrate modification during radiofrequency catheter ablation for atrial fibrillation: a randomized study. Europace. 2018;20(11):1766-75.
41. Kobori A, Shizuta S, Inoue K, Kaitani K, Morimoto T, Nakazawa Y, et al. Adenosine triphosphate-guided pulmonary vein isolation for atrial fibrillation: the UNmasking Dormant Electrical Reconduction by Adenosine TriPhosphate (UNDER-ATP) trial. Eur Heart J. 2015;36(46):3276-87.
42. Kumagai K, Toyama H, Zhang B. Effects of additional ablation of low-voltage areas after Box isolation for persistent atrial fibrillation. J Arrhythm. 2019;35(2):197-204.
43. Lee JM, Shim J, Park J, Yu HT, Kim TH, Park JK, et al. The Electrical Isolation of the Left Atrial Posterior Wall in Catheter Ablation of Persistent Atrial Fibrillation. JACC Clin Electrophysiol. 2019;5(11):1253-61.
44. Lee KN, Choi JI, Kim YG, Oh SK, Kim DH, Lee DI, et al. Comparison between linear and focal ablation of complex fractionated atrial electrograms in patients with non-paroxysmal atrial fibrillation: a prospective randomized trial. Europace. 2019;21(4):598-606.
45. Li X, Bao Y, Jia K, Zhang N, Lin C, Wei Y, et al. Comparison of the Mid-Term Outcomes of Robotic Magnetic Navigation-Guided Radiofrequency Ablation versus Cryoballoon Ablation for Persistent Atrial Fibrillation. J Cardiovasc Dev Dis. 2022;9(3).
46. Lim TW, Koay CH, See VA, McCall R, Chik W, Zecchin R, et al. Single-ring posterior left atrial (box) isolation results in a different mode of recurrence compared with wide antral pulmonary vein isolation on long-term follow-up: longer atrial fibrillation-free survival time but similar survival time free of any atrial arrhythmia. Circ Arrhythm Electrophysiol. 2012;5(5):968-77.
47. Lin R, Zeng C, Xu K, Wu S, Qin M, Liu X. Dispersion-guided ablation in conjunction with circumferential pulmonary vein isolation is superior to stepwise ablation approach for persistent atrial fibrillation. Int J Cardiol. 2019;278:97-103.
48. Lin YJ, Chang SL, Lo LW, Hu YF, Chong E, Chao TF, et al. A prospective and randomized comparison of limited versus extensive atrial substrate modification after circumferential pulmonary vein isolation in nonparoxysmal atrial fibrillation. J Cardiovasc Electrophysiol. 2014;25(8):803-12.
49. Lin YJ, Lo MT, Chang SL, Lo LW, Hu YF, Chao TF, et al. Benefits of Atrial Substrate Modification Guided by Electrogram Similarity and Phase Mapping Techniques to Eliminate Rotors and Focal Sources Versus Conventional Defragmentation in Persistent Atrial Fibrillation. JACC Clin Electrophysiol. 2016;2(6):667-78.
50. Mansour M, Calkins H, Osorio J, Pollak SJ, Melby D, Marchlinski FE, et al. Persistent Atrial Fibrillation Ablation With Contact Force-Sensing Catheter: The Prospective Multicenter PRECEPT Trial. JACC Clin Electrophysiol. 2020;6(8):958-69.
51. Matsuo S, Yamane T, Date T, Hioki M, Narui R, Ito K, et al. Completion of mitral isthmus ablation using a steerable sheath: prospective randomized

comparison with a nonsteerable sheath. J Cardiovasc Electrophysiol. 2011;22(12):1331-8.
52. Mohanty S, Di Biase L, Mohanty P, Trivedi C, Santangeli P, Bai R, et al. Effect of periprocedural amiodarone on procedure outcome in patients with longstanding persistent atrial fibrillation undergoing extended pulmonary vein antrum isolation: results from a randomized study (SPECULATE). Heart Rhythm. 2015;12(3):477-83.
53. Mohanty S, Gianni C, Trivedi C, Metz T, Bai R, Al-Ahmad A, et al. Impact of rotor ablation in non-paroxysmal AF patients: Findings from the per-protocol population of the OASIS trial at long-term follow-up. Am Heart J. 2018;205:145-8.
54. Mont L, Bisbal F, Hernández-Madrid A, Pérez-Castellano N, Viñolas X, Arenal A, et al. Catheter ablation vs. antiarrhythmic drug treatment of persistent atrial fibrillation: a multicentre, randomized, controlled trial (SARA study). Eur Heart J. 2014;35(8):501-7.
55. Mörtsell D, Jansson V, Malmborg H, Lönnerholm S, Blomström-Lundqvist C. Clinical outcome of the 2nd generation cryoballoon for pulmonary vein isolation in patients with persistent atrial fibrillation - A sub-study of the randomized trial evaluating single versus dual cryoballoon applications. Int J Cardiol. 2019;278:120-5.
56. Nery PB, Alqarawi W, Nair GM, Sadek MM, Redpath CJ, Golian M, et al. Catheter Ablation of Low-Voltage Areas for Persistent Atrial Fibrillation: Procedural Outcomes Using High-Density Voltage Mapping. Can J Cardiol. 2020;36(12):1956-64.
57. Onishi N, Kaitani K, Nakagawa Y, Inoue K, Kobori A, Nakazawa Y, et al. The association between late-phase early recurrence within the blanking period after atrial fibrillation catheter ablation and long-term recurrence: Insights from a large-scale multicenter study. Int J Cardiol. 2021;341:39-45.
58. Pachón MJ, Pachón ME, Santillana PT, Lobo TJ, Pachón CTC, Pachón MJ, et al. Ablation of "Background Tachycardia" in Long Standing Atrial Fibrillation: Improving the Outcomes by Unmasking a Residual Atrial Fibrillation Perpetuator. J Atr Fibrillation. 2017;10(2):1583.
59. Panikker S, Jarman JW, Virmani R, Kutys R, Haldar S, Lim E, et al. Left Atrial Appendage Electrical Isolation and Concomitant Device Occlusion to Treat Persistent Atrial Fibrillation: A First-in-Human Safety, Feasibility, and Efficacy Study. Circ Arrhythm Electrophysiol. 2016;9(7).
60. Pavlović N, Sticherling C, Knecht S, Reichlin T, Mühl A, Schaer B, et al. One-year follow-up after irrigated multi-electrode radiofrequency ablation of persistent atrial fibrillation. Europace. 2016;18(1):85-91.
61. Pokushalov E, Romanov A, Katritsis DG, Artyomenko S, Shirokova N, Karaskov A, et al. Ganglionated plexus ablation vs linear ablation in patients undergoing pulmonary vein isolation for persistent/long-standing persistent atrial fibrillation: a randomized comparison. Heart Rhythm. 2013;10(9):1280-6.
62. Rostock T, Salukhe TV, Hoffmann BA, Steven D, Berner I, Müllerleile K, et al. Prognostic role of subsequent atrial tachycardias occurring during ablation of persistent atrial fibrillation: a prospective randomized trial. Circ Arrhythm Electrophysiol. 2013;6(6):1059-65.
63. Sairaku A, Yoshida Y, Kamiya H, Tatematsu Y, Nanasato M, Hirayama H, et al. High-frequency stimulation of the atria increases early recurrence following pulmonary vein isolation in patients with persistent atrial fibrillation. Heart Rhythm. 2012;9(9):1386-92.

64. Sawhney V, Schilling RJ, Providencia R, Cadd M, Perera D, Chatha S, et al. Cryoablation for persistent and longstanding persistent atrial fibrillation: results from a multicentre European registry. Europace. 2020;22(3):375-81.
65. Schmidt B, Neuzil P, Luik A, Osca Asensi J, Schrickel JW, Deneke T, et al. Laser Balloon or Wide-Area Circumferential Irrigated Radiofrequency Ablation for Persistent Atrial Fibrillation: A Multicenter Prospective Randomized Study. Circ Arrhythm Electrophysiol. 2017;10(12).
66. Shi LB, Rossvoll O, Tande P, Schuster P, Solheim E, Chen J. Cryoballoon vs. radiofrequency catheter ablation: insights from NOrwegian randomized study of PERSistent Atrial Fibrillation (NO-PERSAF study). Europace. 2022;24(2):226-33.
67. Singh SM, d'Avila A, Kim YH, Aryana A, Mangrum JM, Michaud GF, et al. The modified stepwise ablation guided by low-dose ibutilide in chronic atrial fibrillation trial (The MAGIC-AF Study). Eur Heart J. 2016;37(20):1614-21.
68. Solheim E, Hoff PI, Off MK, Ohm OJ, Chen J. Significance of late recurrence of atrial fibrillation during long-term follow-up after pulmonary vein isolation. Pacing Clin Electrophysiol. 2007;30 Suppl 1:S108-11.
69. Stabile G, Bertaglia E, Turco P, Zoppo F, Iuliano A, Zerbo F, et al. Role of pulmonary veins isolation in persistent atrial fibrillation ablation: the pulmonary vein isolation in persistent atrial fibrillation (PIPA) study. Pacing Clin Electrophysiol. 2009;32 Suppl 1:S116-9.
70. Su WW, Reddy VY, Bhasin K, Champagne J, Sangrigoli RM, Braegelmann KM, et al. Cryoballoon ablation of pulmonary veins for persistent atrial fibrillation: Results from the multicenter STOP Persistent AF trial. Heart Rhythm. 2020;17(11):1841-7.
71. Takarada K, Ströker E, de Asmundis C, Sieira J, Abugattas JP, Coutiño HE, et al. Second-Generation Cryoballoon Ablation for Atrial Fibrillation - A Detailed Analysis of the Impact of Left Atrial Volume Index on Clinical Outcome. Circ J. 2018;83(1):84-90.
72. Tamborero D, Mont L, Berruezo A, Matiello M, Benito B, Sitges M, et al. Left atrial posterior wall isolation does not improve the outcome of circumferential pulmonary vein ablation for atrial fibrillation: a prospective randomized study. Circ Arrhythm Electrophysiol. 2009;2(1):35-40.
73. Tamborero D, Mont L, Molina I, Matiello M, Berruezo A, Sitges M, et al. Selective segmental ostial ablation and circumferential pulmonary veins ablation. Results of an individualized strategy to cure refractory atrial fibrillation. J Interv Card Electrophysiol. 2007;19(1):19-27.
74. Ullah W, McLean A, Hunter RJ, Baker V, Richmond L, Cantor EJ, et al. Randomized trial comparing robotic to manual ablation for atrial fibrillation. Heart Rhythm. 2014;11(11):1862-9.
75. Valderrábano M, Peterson LE, Swarup V, Schurmann PA, Makkar A, Doshi RN, et al. Effect of Catheter Ablation With Vein of Marshall Ethanol Infusion vs Catheter Ablation Alone on Persistent Atrial Fibrillation: The VENUS Randomized Clinical Trial. Jama. 2020;324(16):1620-8.
76. Verma A, Jiang CY, Betts TR, Chen J, Deisenhofer I, Mantovan R, et al. Approaches to catheter ablation for persistent atrial fibrillation. N Engl J Med. 2015;372(19):1812-22.
77. Verma A, Mantovan R, Macle L, De Martino G, Chen J, Morillo CA, et al. Substrate and Trigger Ablation for Reduction of Atrial Fibrillation (STAR AF): a randomized, multicentre, international trial. Eur Heart J. 2010;31(11):1344-56.

78. Verma A, Patel D, Famy T, Martin DO, Burkhardt JD, Elayi SC, et al. Efficacy of adjuvant anterior left atrial ablation during intracardiac echocardiography-guided pulmonary vein antrum isolation for atrial fibrillation. J Cardiovasc Electrophysiol. 2007;18(2):151-6.
79. Verma A, Sanders P, Champagne J, Macle L, Nair GM, Calkins H, et al. Selective complex fractionated atrial electrograms targeting for atrial fibrillation study (SELECT AF): a multicenter, randomized trial. Circ Arrhythm Electrophysiol. 2014;7(1):55-62.
80. Vogler J, Willems S, Sultan A, Schreiber D, Lüker J, Servatius H, et al. Pulmonary Vein Isolation Versus Defragmentation: The CHASE-AF Clinical Trial. J Am Coll Cardiol. 2015;66(24):2743-52.
81. Wang M, Zhao Q, Ding W, Cai S. Comparison of Direct Current Synchronized Cardioversion to Ibutilide-Guided Catheter Ablation for Long-Term Sinus Rhythm Maintenance After Isolated Pulmonary Vein Isolation of Persistent Atrial Fibrillation. Am J Cardiol. 2017;119(12):1997-2002.
82. Wang XH, Li Z, Mao JL, He B. A novel individualized substrate modification approach for the treatment of long-standing persistent atrial fibrillation: preliminary results. Int J Cardiol. 2014;175(1):162-8.
83. Wang YL, Liu X, Tan HW, Zhou L, Jiang WF, Gu J, et al. Evaluation of linear lesions in the left and right atrium in ablation of long-standing atrial fibrillation. Pacing Clin Electrophysiol. 2013;36(10):1202-10.
84. Wang YL, Liu X, Zhang Y, Jiang WF, Zhou L, Qin M, et al. Optimal endpoint for catheter ablation of longstanding persistent atrial fibrillation: A randomized clinical trial. Pacing Clin Electrophysiol. 2018;41(2):172-8.
85. Willems S, Klemm H, Rostock T, Brandstrup B, Ventura R, Steven D, et al. Substrate modification combined with pulmonary vein isolation improves outcome of catheter ablation in patients with persistent atrial fibrillation: a prospective randomized comparison. Eur Heart J. 2006;27(23):2871-8.
86. Willems S, Verma A, Betts TR, Murray S, Neuzil P, Ince H, et al. Targeting Nonpulmonary Vein Sources in Persistent Atrial Fibrillation Identified by Noncontact Charge Density Mapping: UNCOVER AF Trial. Circ Arrhythm Electrophysiol. 2019;12(7):e007233.
87. Wong KC, Paisey JR, Sopher M, Balasubramaniam R, Jones M, Qureshi N, et al. No Benefit of Complex Fractionated Atrial Electrogram Ablation in Addition to Circumferential Pulmonary Vein Ablation and Linear Ablation: Benefit of Complex Ablation Study. Circ Arrhythm Electrophysiol. 2015;8(6):1316-24.
88. Wu G, Huang H, Cai L, Yang Y, Liu X, Yu B, et al. Long-term observation of catheter ablation vs. pharmacotherapy in the management of persistent and long-standing persistent atrial fibrillation (CAPA study). Europace. 2021;23(5):731-9.
89. Wynn GJ, Panikker S, Morgan M, Hall M, Waktare J, Markides V, et al. Biatrial linear ablation in sustained nonpermanent AF: Results of the substrate modification with ablation and antiarrhythmic drugs in nonpermanent atrial fibrillation (SMAN-PAF) trial. Heart Rhythm. 2016;13(2):399-406.
90. Xu G, Cai J, Liu Z, Liu E, Jing X, Liu T, et al. Clinical efficacy of "ICE-FIRE" ablation for non-paroxysmal atrial fibrillation. J Interv Card Electrophysiol. 2021;60(2):205-11.
91. Yao Y, Hu F, Du Z, He J, Shi H, Zhang J, et al. The value of extensive catheter linear ablation on persistent atrial fibrillation (the CLEAR-AF Study). Int J Cardiol. 2020;316:125-9.

92. Yoshiga Y, Shimizu A, Ueyama T, Ono M, Fukuda M, Fumimoto T, et al. Strict sequential catheter ablation strategy targeting the pulmonary veins and superior vena cava for persistent atrial fibrillation. J Cardiol. 2018;72(2):128-34.
93. Choi Y, Lim B, Yang SY, Yang SH, Kwon OS, Kim D, et al. Clinical Usefulness of Virtual Ablation Guided Catheter Ablation of Atrial Fibrillation Targeting Restitution Parameter-Guided Catheter Ablation: CUVIA-REGAB Prospective Randomized Study. Korean Circ J. 2022;52(9):699-711.
94. Kistler PM, Chieng D, Sugumar H, Ling L-H, Segan L, Azzopardi S, et al. Effect of Catheter Ablation Using Pulmonary Vein Isolation With vs Without Posterior Left Atrial Wall Isolation on Atrial Arrhythmia Recurrence in Patients With Persistent Atrial Fibrillation: The CAPLA Randomized Clinical Trial. JAMA. 2023;329(2):127-35.
95. Li K, Xu C, Zhu X, Wang X, Ye P, Jiang W, et al. Multi-centre, prospective randomized comparison of three different substrate ablation strategies for persistent atrial fibrillation. Europace. 2023;25(5).
96. Marrouche NF, Wazni O, McGann C, Greene T, Dean JM, Dagher L, et al. Effect of MRI-Guided Fibrosis Ablation vs Conventional Catheter Ablation on Atrial Arrhythmia Recurrence in Patients With Persistent Atrial Fibrillation: The DECAAF II Randomized Clinical Trial. Jama. 2022;327(23):2296-305.
97. Verma A, Haines DE, Boersma LV, Sood N, Natale A, Marchlinski FE, et al. Pulsed Field Ablation for the Treatment of Atrial Fibrillation: PULSED AF Pivotal Trial. Circulation. 2023;147(19):1422-32.
98. Yang G, Zheng L, Jiang C, Fan J, Liu X, Zhan X, et al. Circumferential Pulmonary Vein Isolation Plus Low-Voltage Area Modification in Persistent Atrial Fibrillation: The STABLE-SR-II Trial. JACC Clin Electrophysiol. 2022;8(7):882-91.
99. Huo Y, Gaspar T, Schönbauer R, Wójcik M, Fiedler L, Roithinger FX, et al. Low-Voltage Myocardium-Guided Ablation Trial of Persistent Atrial Fibrillation. NEJM Evidence. 2022;0(0):EVIDoa2200141.
